# In Vivo Inflammation Caused by *Achromobacter* spp. Cystic Fibrosis Clinical Isolates Exhibiting Different Pathogenic Characteristics

**DOI:** 10.3390/ijms24087432

**Published:** 2023-04-18

**Authors:** Angela Sandri, Giulia Maria Saitta, Laura Veschetti, Federico Boschi, Rebeca Passarelli Mantovani, Maria Carelli, Paola Melotti, Caterina Signoretto, Marzia Boaretti, Giovanni Malerba, Maria M. Lleò

**Affiliations:** 1 Department of Diagnostics and Public Health, Microbiology Section, University of Verona, 37134 Verona, Italy; 2 GMLab, Department of Neurosciences, Biomedicine and Movement Sciences, University of Verona, 37134 Verona, Italy; 3 Department of Engineering for Innovation Medicine, University of Verona, 37134 Verona, Italy; 4 Cystic Fibrosis Center, Azienda Ospedaliera Universitaria Integrata Verona, 37126 Verona, Italy

**Keywords:** *Achromobacter*, virulence, cytotoxicity, biofilm, acute respiratory infection, lung inflammation

## Abstract

*Achromobacter* spp. lung infection in cystic fibrosis has been associated with inflammation, increased frequency of exacerbations, and decline of respiratory function. We aimed to evaluate in vivo the inflammatory effects of clinical isolates exhibiting different pathogenic characteristics. Eight clinical isolates were selected based on different pathogenic characteristics previously assessed: virulence in *Galleria mellonella* larvae, cytotoxicity in human bronchial epithelial cells, and biofilm formation. Acute lung infection was established by intratracheal instillation with 10.5 × 10^8^ bacterial cells in wild-type and CFTR-knockout (KO) mice expressing a luciferase gene under control of interleukin-8 promoter. Lung inflammation was monitored by in vivo bioluminescence imaging up to 48 h after infection, and mortality was recorded up to 96 h. Lung bacterial load was evaluated by CFU count. Virulent isolates caused higher lung inflammation and mice mortality, especially in KO animals. Isolates both virulent and cytotoxic showed higher persistence in mice lungs, while biofilm formation was not associated with lung inflammation, mice mortality, or bacterial persistence. A positive correlation between virulence and lung inflammation was observed. These results indicate that *Achromobacter* spp. pathogenic characteristics such as virulence and cytotoxicity may be associated with clinically relevant effects and highlight the importance of elucidating their mechanisms.

## 1. Introduction

Cystic Fibrosis (CF) is a genetic disorder caused by mutations in the cystic fibrosis transmembrane regulator (CFTR) gene, encoding for CFTR protein, an important chloride channel of exocrine glands [1]. Although CF is a multisystemic disease, 85% of the mortality results from lung impairment [2]. Lack of functional CFTR expression in the airways results in excessive thickening of the mucus layer, which favors the development of infections by a large number of opportunistic pathogens such as *Staphylococcus aureus*, *Pseudomonas aeruginosa*, *Burkholderia cepacia* complex, *Stenotrophomonas maltophilia*, and *Achromobacter* spp. [3]. These bacteria are able to colonize persistently the lungs of people with CF, influencing the course of the disease, usually with decline of lung function and increased risk of mortality. Among them, in the last decade, *Achromobacter* spp. have generated growing interest as emerging opportunistic pathogens in CF that can cause severe chronic infections leading to lung inflammation, increased frequency of exacerbation, and decline of the respiratory function [4].

The pathogenic mechanisms of many CF pathogens are nowadays well known; in particular, *P. aeruginosa* has been extensively studied, due to its high incidence in CF patients and difficult eradication. Conversely, little is known about the pathogenicity of *Achromobacter* spp. Similar to other CF-related pathogens such as *P. aeruginosa*, a number of virulence factors have already been described in *Achromobacter* spp., such as production of secreted factors (e.g., colicin V and proteases), expression of membrane-bound factors (e.g., Vi capsular polysaccharide and O-antigen), secretion systems (e.g., type II and III secretion systems), and biofilm formation, which are important for survival and proliferation in hostile environments [5,6,7,8]. Moreover, these bacteria show innate and acquired resistance to many classes of antibiotics, especially to aminoglycosides, aztreonam, tetracyclines, penicillins, cephalosporins, and β-lactams [9]. Nonetheless, the pathogenic mechanisms and virulence features connected to *Achromobacter* spp. capability of colonizing CF lungs chronically or occasionally are still unclear.

In a previous study, we observed that *Achromobacter* spp. clinical isolates can exhibit different pathogenic characteristics (e.g., virulence, cytotoxicity, biofilm formation) that could be associated with different clinical outcomes [10]. Lung inflammation has already been reported to be influenced by bacterial virulence for another CF pathogen, *P. aeruginosa* [11]. We hypothesized that a similar situation could exist and be relevant for *Achromobacter* spp. too, and evaluated in vivo the inflammatory effects of *Achromobacter* spp. clinical isolates presenting different pathogenic characteristics.

## 2. Results

Wild-type (WT) and CFTR-knockout (KO) mice were intratracheally challenged with 8 *Achromobacter* spp. clinical isolates expressing different pathogenic characteristics: virulence in *G. mellonella* larvae, cytotoxicity in bronchial epithelial cells, and biofilm formation previously assessed [10], as shown in Table 1 and defined in Tables S1–S3. Mice were grouped and analyzed based on the pathogenic characteristics of the isolates. For each characteristic, strains were divided into 2 groups: high–medium and low–no activity.

### 2.1. Lung Inflammation Induced by Isolates Exhibiting Different Pathogenic Characteristics

**Figure 1:**
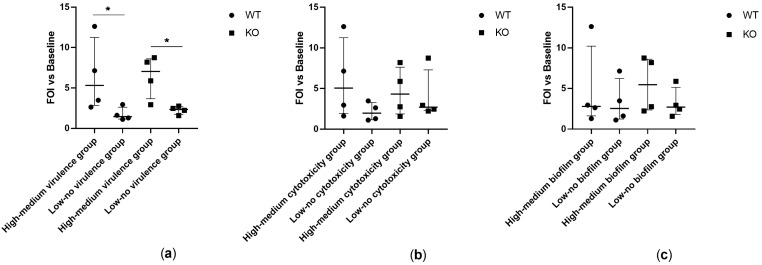
IL-8-dependent bioluminescence emitted by WT and KO mice at 24 h after intratracheal challenge with clinical isolates showing (**a**) high–medium vs. high-medium virulence in *G. mellonella* larvae, (**b**) high–medium vs. low–no cytotoxicity in human bronchial epithelial cells, and (**c**) high–medium vs. low–no biofilm formation. Photon emission is expressed as Folds of Induction (FOI) vs. baseline (before lung challenge). Each value represents the mean of four animals challenged with each isolate. The median ± interquartile range of 16 mice per group is shown. Statistical analysis was performed by Kruskal–Wallis test; * *p* < 0.05.

**Figure 2:**
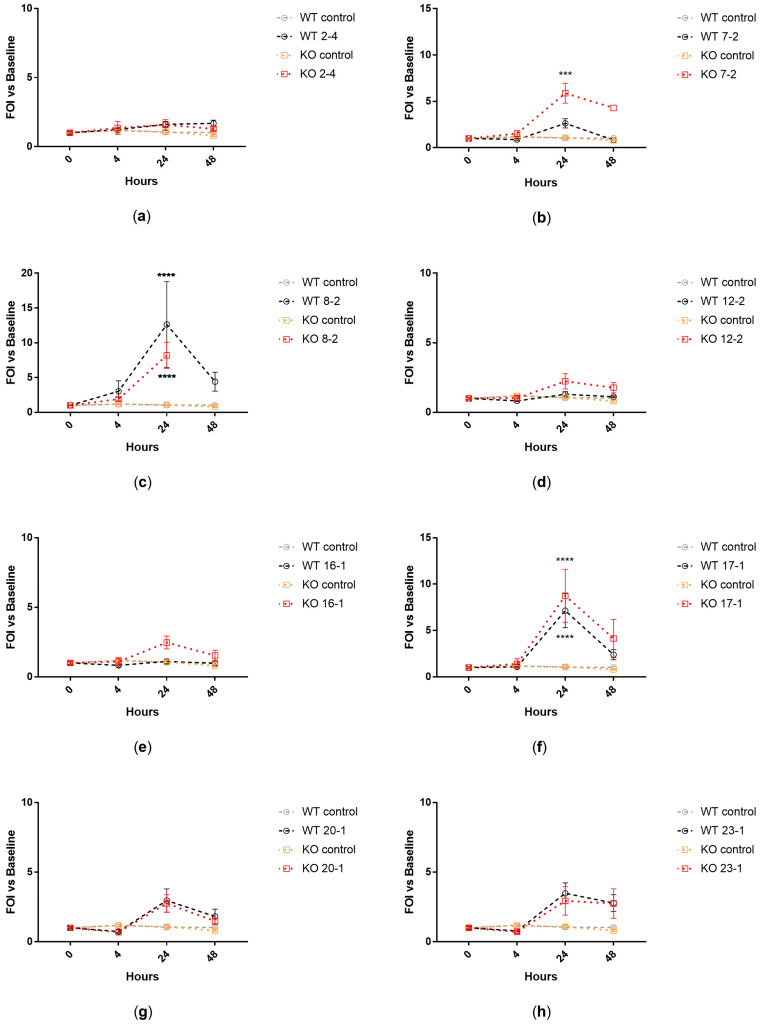
IL-8-dependent bioluminescence emitted by WT and KO mice after intratracheal challenge with each clinical isolate: 2-4 (**a**), 7-2 (**b**), 8-2 (**c**), 12-2 (**d**), 16-1 (**e**), 17-1 (**f**), 20-1 (**g**), 23-1 (**h**). Imaging was performed before lung challenge (0 h) and after 4, 24, and 48 h. FOI = Folds of Induction vs. baseline (0 h). Each value represents the mean ± SEM of four animals. Statistical analysis of treated vs. control mice was performed by repeated measures 2-way ANOVA followed by Tukey’s multiple comparison test; *** *p* < 0.001, **** *p* < 0.0001.

Transgenic WT and KO mice expressing a luciferase gene under control of bovine interleukin-8 (IL-8) promoter were intratracheally challenged with *Achromobacter* spp. clinical isolates. IL-8-dependent lung inflammation was monitored by in vivo bioluminescence imaging after 4, 24, and 48 h. Isolates showing high–medium virulence in larvae induced significantly higher IL-8-dependent bioluminescence emission in both WT (*p* = 0.026) and KO (*p* = 0.038) mice in comparison to isolates with low–no virulence (Figure 1). When analyzing the isolates individually, three out of four strains showing high–medium virulence activity induced a significant increase of IL-8-dependent bioluminescence emission in both WT and KO mice (isolates 8-2, 17-1), or in KO mice only (isolate 7-2), in comparison to control mice (Figure 2).

### 2.2. Mice Survival during Acute Infection with Isolates Exhibiting Different Pathogenic Characteristics

**Figure 3:**
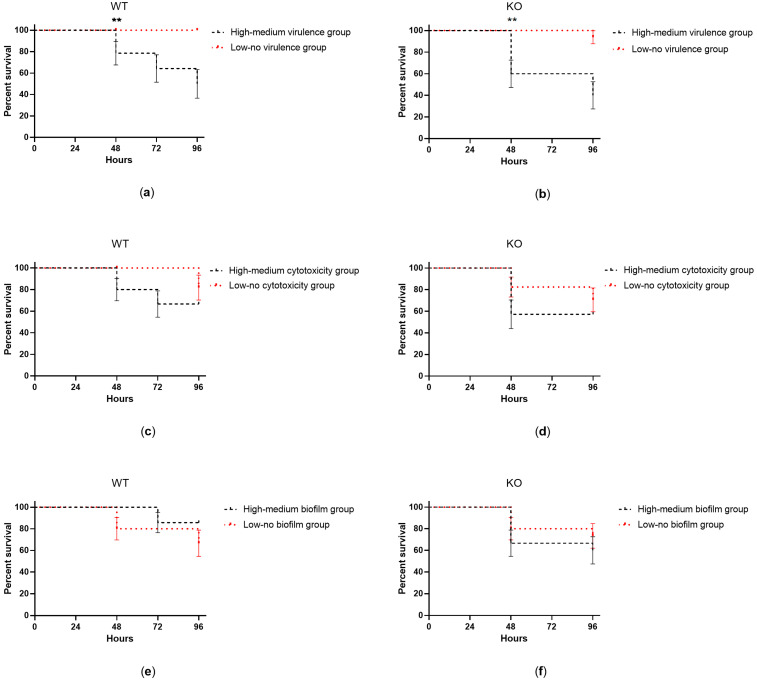
Percent survival of WT and KO mice up to 96 h after intratracheal challenge with clinical isolates showing (**a**,**b**) high–medium vs. low–no virulence in *G. mellonella* larvae, (**c**,**d**) high–medium vs. low–no cytotoxicity in human bronchial epithelial cells, and (**e**,**f**) high–medium vs. low–no biofilm formation. The mean ± SEM of 16 mice per group (*n* = 4 treated with each isolate) is shown. Statistical analysis was performed by log-rank test; ** *p* < 0.01.

**Figure 4:**
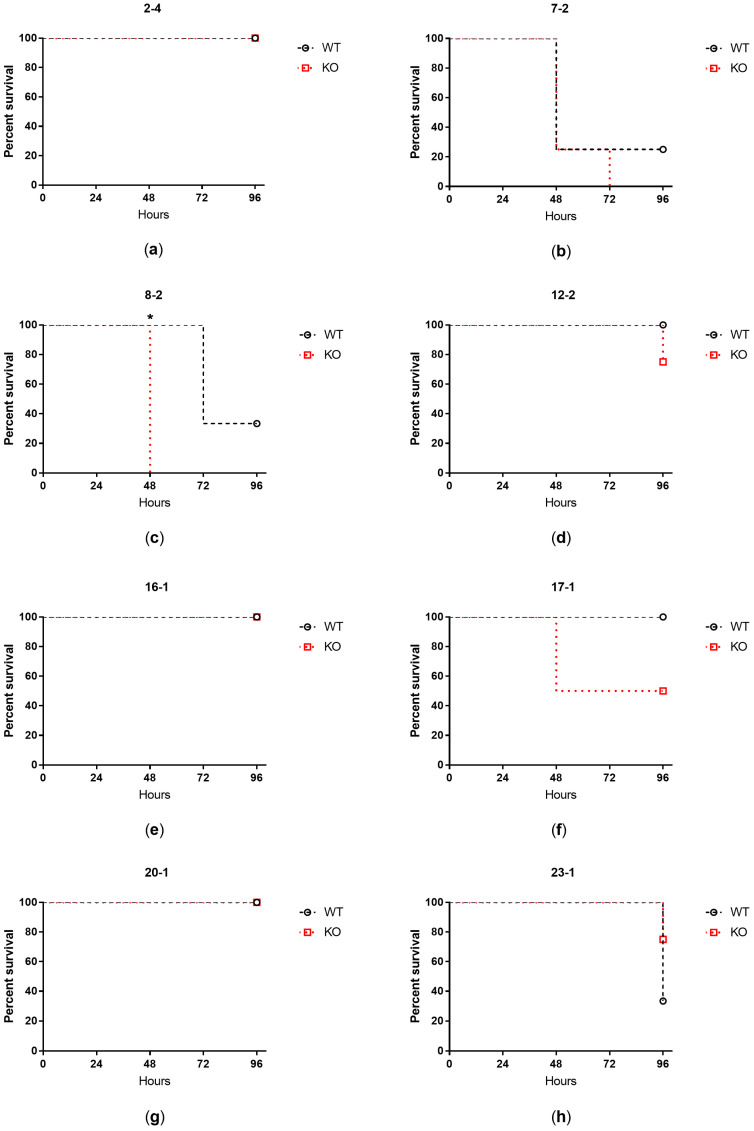
Percent survival of WT and KO mice up to 96 h after intratracheal challenge with each clinical isolate: 2-4 (**a**), 7-2 (**b**), 8-2 (**c**), 12-2 (**d**), 16-1 (**e**), 17-1 (**f**), 20-1 (**g**), 23-1 (**h**). The mean of four mice per group is shown. Statistical analysis was performed by log-rank test; * *p* < 0.05.

Mice survival was monitored up to 96 h from the intratracheal challenge with the clinical isolates. Isolates showing high–medium virulence in larvae induced significantly higher mortality in both WT (*p* = 0.0035) and KO (*p* = 0.0012) mice in comparison to isolates with low–no virulence (Figure 3). When analyzing the isolates individually, two strains showing high–medium virulence activity caused over 65% mortality within 72 h from lung challenge in both WT and KO mice (isolates 7-2, 8-2) (Figure 4). One of them (isolate 8-2) induced significantly faster mortality in KO mice (24 h) than in WT animals (48 h) (Figure 4c).

### 2.3. Bacterial Load during Acute Infection with Isolates Exhibiting Different Pathogenic Characteristics

**Figure 5:**
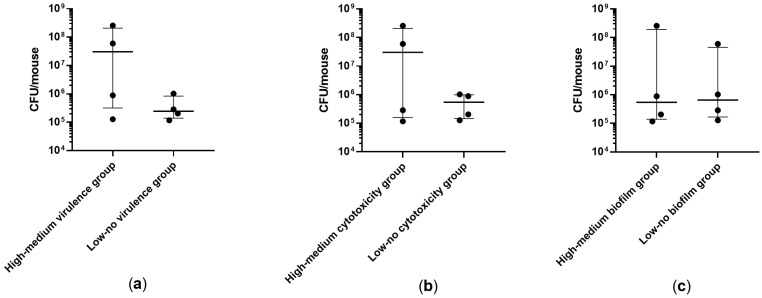
CFU recovered from WT mice lungs at 24 h after intratracheal challenge with clinical isolates showing (**a**) high–medium vs. low–no virulence in *G. mellonella* larvae, (**b**) high–medium vs. low–no cytotoxicity in human bronchial epithelial cells, (**c**) high–medium vs. low–no biofilm formation. Each value represents the mean of three animals challenged with each isolate. The median ± interquartile range of 12 mice per group is shown.

**Figure 6:**
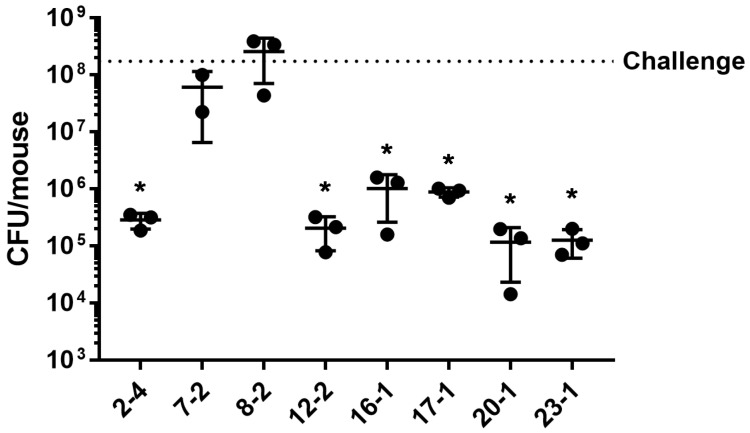
CFU recovered from WT mice lungs at 24 h after intratracheal challenge with eight clinical isolates. The bacterial load used for the challenge is indicated (dotted line). The mean ± SD of three mice per group is shown. Statistical analysis of each isolate vs. challenge was performed by 1way ANOVA test; * *p* < 0.05.

Bacterial load in the lungs was evaluated after 24 h from the intratracheal challenge with the clinical isolates. For all the three pathogenic characteristics analyzed, no significant difference was observed between isolates exhibiting high–medium and low–no activity (Figure 5). Nonetheless, when analyzing the isolates individually, after 24 h from the lung challenge, two isolates with both high–medium virulence and cytotoxicity (7-2, 8-2) showed a high bacterial load in the mice lungs comparable to the challenging dosage, indicating that no clearance occurred, while the bacterial load of all the other isolates was significantly reduced in comparison to the challenging dosage (Figure 6).

### 2.4. Lung Inflammation in Mice Correlates with Virulence in G. mellonella Larvae

**Figure 7:**
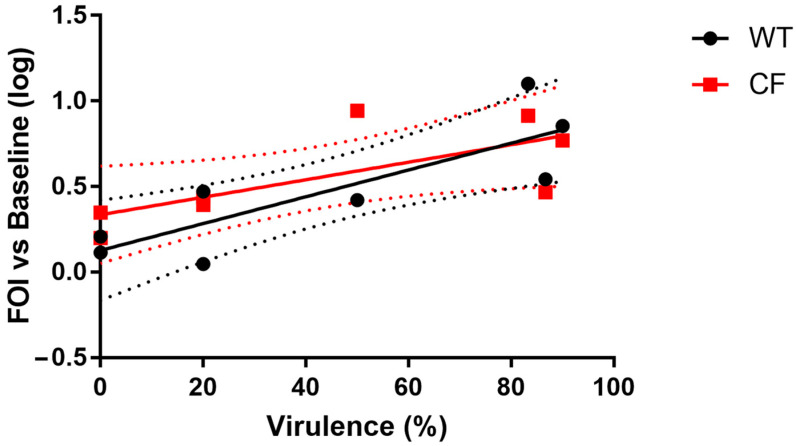
Correlation between IL-8-dependent bioluminescence emission in WT and KO mice at 24 h after intratracheal challenge with the clinical isolates and virulence in *G. mellonella* larvae infected with the same isolates. FOI = Fold of Induction vs. baseline (before lung challenge). Each point represents the mean ± SEM of four mice challenged with each isolate. Linear regression ± SEM (dotted lines) is shown.

Based on results from IL-8-dependent bioluminescence emission, mice survival, and lung bacterial load, we identified virulence as the most interesting pathogenic characteristic among those analyzed. We found a positive correlation between virulence of the isolates in *G. mellonella* larvae and IL-8-dependent bioluminescence emission at 24 h after intratracheal challenge in both WT and KO mice (*p* = 0.027 in WT mice, *p* = 0.032 in KO mice; Spearman r = 0.78 in WT mice, r = 0.77 in KO mice) (Figure 7).

## 3. Discussion

*Achromobacter* spp. exhibit different pathogenic characteristics that might be linked to clinical outcomes in CF lung infection. We previously evaluated the pathogenicity of *Achromobacter* spp. in terms of virulence in *G. mellonella* larvae, cytotoxicity in human bronchial epithelial cells, and biofilm formation, and observed wide differences for each of these three characteristics among clinical isolates [10]. Based on these results, in the present study, we selected eight isolates showing different pathogenic characteristics and used them to induce acute lung infection in WT and KO mice to observe eventual differences in lung inflammation, mice survival, and bacterial load that could reveal the importance of these characteristics for *Achromobacter* spp. pathogenicity in CF lung infection.

We found a significant positive correlation between *Achromobacter* spp. virulence in *G. mellonella* larvae and in mice (both WT and KO). In particular, virulent isolates inducing high larvae mortality also induced high lung inflammation and mortality in mice. A correlation between mortality in mice and larvae had been reported for *P. aeruginosa* PA14 mutants [13], indicating that insect model systems can provide a tool for the characterization of microbial virulence involved in causing diseases in mammals. Our results further confirm that *G. mellonella* can be a useful infection model to study virulence of CF respiratory pathogens, also related to virulence-induced inflammation.

There are a number of virulence factors that could be involved in pathogenicity in both larvae and mice, from extracellular proteases to exotoxins to lipopolysaccharide, which were reported to exhibit pathogenicity in both models when treated with *P. aeruginosa* [11,13,14,15,16]. Our previous genomic profiling of the isolates used in this study shows that three (out of four) of the less virulent isolates (i.e., 16-1, 12-2, 20-1) also lack several genes associated with the ability to infect cells [12], chemotactic movement, type 3 secretion system, and proteases. This could explain both their low virulence in the *G. mellonella* model, and the low inflammation and mortality observed in mice.

Interestingly, only two isolates expressing both cytotoxicity and virulence showed a higher bacterial load in the mouse lungs after 24 h from the infection procedure, suggesting a higher persistence ability, while all the other strains lacked the combination of both characteristics (virulence and cytotoxicity) and resulted in a bacterial load significantly lower than the challenging dosage. This suggests that both cytotoxicity and virulence could be necessary for successful colonization of the lung environment by *Achromobacter* spp.

Conversely, biofilm formation was not relevant for the induction of lung inflammation and mice mortality, nor for the maintenance of a high bacterial load. The latter might seem surprising at first, since biofilm has been documented as an important mechanism of infection for many pathogens in several diseases (e.g., infective endocarditis, otitis media, urinary tract infections, etc.) including CF [17,18]. However, *Achromobacter* spp.’s poor adhesion ability is already known [5,6], suggesting that these bacteria rely on other mechanisms for colonization. For instance, various studies highlighted *Achromobacter* spp.’s ability to form unattached or loosely attached aggregates held together by polysaccharides and characterized by the scattering and dispersal of planktonic cells [19,20].

Few differences associated with the only two isolates expressing both cytotoxicity and virulence were observed between WT and KO animals in our study in terms of higher IL-8-dependent bioluminescence emission and faster mortality in KO than in WT animals. Therefore, the KO mice seem to be more sensitive to *Achromobacter* spp. pathogenicity than the congenic WT animals; this is as expected, considering that the C57BL/6J Cftr^tm1UNC^ strain was reported to consistently develop various aspects of spontaneous and progressive lung disease [21]. Many studies have proved the suitability of this animal model to study induced CF lung infection and inflammation with various microorganisms [22,23,24,25,26,27,28,29]; our study confirms its suitability to study *Achromobacter* spp. lung infection.

Our study is limited to a small number of clinical isolates that could be used in vivo. Using a larger number of strains, each exhibiting only one specific pathogenic characteristic among the three analyzed (virulence, cytotoxicity, and biofilm formation), would have been preferable. However, very few strains with this characteristic were available in our collection. As such, we decided to group the eight strains used in the study based on each pathogenic characteristic, obtaining an equal number of strains (*n* = 4) in each group and ensuring that the same isolates were never grouped together for more than one characteristic (virulence, cytotoxicity, biofilm), as shown in Table 1. This strategy allowed us to evaluate all three characteristics, even using a low number of isolates. Further studies are encouraged to elucidate the extent of the correlations that we have first observed.

In conclusion, our results indicate that *Achromobacter* spp. Pathogenicity may be associated with increased lung inflammation, mortality, and bacterial persistence in vivo. In particular, virulence in terms of *G. mellonella* larvae mortality correlates with pro-inflammatory effects and, coupled with cytotoxicity, could support *Achromobacter* spp.’s persistence. This highlights the importance of elucidating the mechanisms underlying *Achromobacter* spp. pathogenicity and virulence and suggests that the treatment of virulent isolates at an early stage of the infection could help prevent the worsening of the lung disease.

## 4. Materials and Methods

### 4.1. Clinical Isolates

Eight *Achromobacter* spp. clinical isolates were collected from the sputum samples of eight patients at the CF Center of Verona, Italy. All the isolates were identified by whole genome sequencing and phylogenetic analysis, as previously reported [12]. Strains were stored in Microbank (Pro-Lab Diagnostics, Neston, UK) at −80 °C.

For data analysis, the eight clinical isolates were divided in groups (*n* = 4 per group) based on each of the three pathogenic characteristics previously assessed in vitro: virulence in *G. mellonella* larvae, cytotoxicity on human bronchial epithelial cells, and biofilm formation [10]. Briefly, virulence was tested through inoculation of bacteria in *G. mellonella* larvae; in vitro cytotoxicity was tested by quantitative measurement of lactate dehydrogenase in human bronchial epithelial cells; biofilm production was measured by crystal violet staining of surface-attached bacteria cultured in static conditions [30]. Cut-off values were defined, and each strain was assigned a level of activity (high, medium, low, none) for virulence, cytotoxicity, and biofilm formation, as shown in Tables S1–S3, respectively. For each characteristic, strains were divided in two groups: high–medium and low–no activity.

### 4.2. Experimental Animals

Female 12-weeks old congenic C57BL/6NCrlBR (WT) and C57BL/6NCrlBR Cftr^tm1UNC^TgN(FABPCFTR) [21,31] (KO) mice were provided by the Cystic Fibrosis Animal Core facility (San Raffaele Hospital, Milan, Italy) and bred by Charles River (Calco, Lecco, Italy). Animals were maintained under conventional housing conditions. Prior to use, animals were acclimatized for at least 7 days to the local vivarium conditions, having free access to standard rodent chow and tap water. Animal experiments were conducted in compliance with national (Legislative Decree 26/2014, authorization no. 265/2020-PR) and international laws and policies (Guide for the Care and Use of Laboratory Animals).

### 4.3. Reporter Construct

Experimental animals were transfected with the bovine interleukin-8 promoter/luciferase (bIL-8-Luc) construct, containing a luciferase gene under the control of the bovine IL-8 promoter (kindly provided by Professor Gaetano Donofrio, University of Parma, Italy) [32]. Competent *Escherichia coli* DH5α cells were transformed by heat shock and the plasmid was purified by Qiagen Plasmid Plus Mega Kit (Qiagen, cat. no. 12981; Qiagen, Valencia, CA, USA). Plasmid concentration and purity were evaluated using NanoDrop 2000c spectrophotometer (Thermo Fisher Scientific, Fremont, CA, USA).

### 4.4. In Vivo Gene Delivery

In vivo JetPEI (Polyplus Transfection, Illkirch-Graffenstaden, France) was used as carrier for delivering bIL-8-Luc construct to lung tissue. As previously described [32], DNA and JetPEI were mixed with a final nitrogen/phosphate (N/P) ratio of 7–7.5 following the manufacturer’s instructions. Briefly, 38–42 µg DNA and 50.3–60.3 µL JetPEI were separately diluted in 200 µL 5% glucose, mixed, and incubated at room temperature for 15 min; 400 µL of the mixture was intravenously injected through the tail vein after warming the animals for 5 min under a heating lamp. Expression and inactivation of the reporter were monitored by in vivo bioluminescence imaging after 24 h and 7 days, respectively.

### 4.5. Bacterial Preparation for Lung Challenge

*Achromobacter* spp. strains were plated onto Luria–Bertani (LB) agar (Oxoid, Basingstoke, UK) and grown at 37 °C for 24–48 h. A single colony was inoculated in LB medium and grown for 16 h at 37 °C shaking. Bacteria were washed twice and resuspended in saline solution. Absorbance at 600 nm was measured, and bacterial cells were diluted to the appropriate load for lung challenge.

### 4.6. Intratracheal Instillation

BIL-8-Luc transgenic mice were intratracheally challenged with 1.5 × 10^8^ CFU of *Achromobacter* spp. cells for the development of acute lung infection [33]. As previously described [32], mice were anesthetized with 2.5% isoflurane and placed on an intubation platform, hanging by their incisor teeth. After visualization of the opening of the trachea using a laryngoscope, 50 µL of bacterial suspension was instilled by an intubation tube connected to a pressure control system. After 4, 24, and 48 h, reporter activation was monitored by in vivo imaging. Control non-infected mice were intratracheally instilled with saline solution. All mice were also imaged before the intratracheal instillation (baseline).

### 4.7. In Vivo Bioluminescence Imaging

Bioluminescence imaging of experimental animals was performed, as previously described [32], using IVIS Spectrum imaging system (Xenogen, Alameda, CA, USA). Ten minutes prior to bioluminescence recording, mice were anesthetized with 2.5% isoflurane and intraperitoneally injected with 150 mg/kg D-Luciferin (PerkinElmer, cat. n. 122,799). After 5 min-long luminescence recording, the photons flux emitted from the chest region was quantified using Living Image 4.7 software (PerkinElmer, Milan, Italy).

### 4.8. Lung Recovery and CFU Count

Mice were euthanized by cervical dislocation and lungs were excised and homogenized using the gentleMACS™ Dissociator (Miltenyi Biotec, Bologna, Italy) according to the manufacturer’s instructions. Briefly, lungs were placed in 2 mL sterile saline solution within a M tube (Miltenyi Biotec, Bologna, Italy) and processed twice using the RNA_01 program. Homogenate was plated on LB agar. Plates were incubated at 37 °C for 24–48 h and CFU were counted.

### 4.9. Statistical Analysis

Statistical analysis was performed using GraphPad Prism version 7.0 software. Mice bioluminescence emission was analyzed by repeated measures 2-way ANOVA followed by Tukey’s multiple comparison test. Mice bioluminescence between the different groups was analyzed by Kruskal–Wallis test. Mice survival was analyzed by log-rank test. CFU between different groups were analyzed by Mann–Whitney test. CFU among clinical isolates were analyzed by 1-way ANOVA. Linear regression and Spearman correlation of mice bioluminescence vs. larvae mortality induced by the clinical isolates were calculated.

## Figures and Tables

**Table 1:** Type of infection of the clinical isolates and levels of activity for each pathogenic characteristic.

Isolate	Species	Virulence	Cytotoxicity	Biofilm
2-4	*A. xylosoxidans*	No	Medium	Low
7-2	*A. xylosoxidans*	High	Medium	Low
8-2	*A. xylosoxidans*	High	Medium	High
12-2	*A. agrifaciens*	No	Low	High
16-1	na	No	Low	Low
17-1	*A. xylosoxidans*	Medium	No	High
20-1	*A. insolitus*	No	Medium	Medium
23-1	*A. xylosoxidans*	High	Low	Low

na = not available. Average nucleotide identity < 95% against all available genomic assemblies used for phylogenetic analysis [12].

## Data Availability

Data are available upon reasonable request to the corresponding author.

## Supplementary Materials

The following supporting information can be downloaded at: https://www.mdpi.com/article/10.3390/ijms24087432/s1.
