# Supplementary material for: In Vivo Inflammation Caused by Achromobacter spp. Cystic Fibrosis Clinical Isolates Exhibiting Different Pathogenic Characteristics
Source: Int J Mol Sci. 2023 Apr 18;24(8):7432. doi: 10.3390/ijms24087432 (PMC10139000; doi:10.3390/ijms24087432)
Supplement: Supplementary file 1 [file ijms-24-07432-s001.zip › ijms-2296284-supplementary.pdf]

Supplementary Tables

Table S1. Virulence of the clinical isolates in *Galleria mellonella* larvae. Ten larvae were inoculated with 1×10^6 bacterial cells of each clinical isolate and monitored daily up to 72 h until death was assessed. Virulence levels, from high to none, were defined as follows: high - with at least 50% mortality in 24 hours; medium – with at least 50% mortality in 48 hours; low - with at least 50% mortality in 72 hours; none – with less than 50% mortality in 72 hours. For each isolate, the number of deaths at each time point is shown and the corresponding level of virulence is indicated.

	Virulence			
**Isolate**	**24h**	**48h**	**72h**	**Level**
**2-4**	0	0	0	No
**7-2**	7	3	0	High
**8-2**	5	5	0	High
**12-2**	0	0	0	No
**16-1**	2	0	0	No
**17-1**	1	4	4	Medium
**20-1**	2	0	0	No
**23-1**	6	4	0	High

Table S2. Cytotoxicity of the clinical isolates in human bronchial epithelial cells. Human CF bronchial epithelial CFBE14o- 4.7 WT-CFTR (WT) and DeltaF508-CFTR (CF) cells were treated with 1×10^8 bacterial cells of each isolate for 4 hours. LDH release was measured using CytoTox 96 Non-Radioactive Cytotoxicity Assay (Promega) according to manufacturer's instructions. Optical density (OD) at 450 nm was recorded and cytotoxicity was calculated by dividing for the absorbance of the positive control. Cytotoxicity levels, from none to high, were defined as follows. The lowest cut-off (0.18) to discriminate no-to-low levels was calculated as one standard deviation (SD) above the mean OD of the negative control; 2- and 4-folds values were used to discriminate between the other levels (low-to-medium = 0.36; medium-to-high = 0.54). For each isolate, the cytotoxicity data in WT and CF cells are shown, and the corresponding level of cytotoxicity is indicated.

	Cytotoxicity		
**Isolate**	**WT**	**CF**	**Level**
**2-4**	0.42	0.41	Medium
**7-2**	0.41	0.35	Medium
**8-2**	0.44	0.48	Medium
**12-2**	0.31	0.39	Low
**16-1**	0.18	0.12	No
**17-1**	0.17	0.16	No
**20-1**	0.49	0.37	Medium
**23-1**	0.21	0.21	Low

Table S3. Biofilm formation ability of the clinical isolates. Biofilm formation was measured by culturing 2×10^7 bacterial cells of each isolate in BHI medium in static conditions at 37°C for 16 hours. Surface-attached cells were stained with 0.1% crystal violet and absorbance at 550 nm was measured. Biofilm levels, from none to high, were defined as proposed by Stepanovic and colleagues [30]. The lowest cut-off (0.03) to discriminate no-to-low levels was calculated as three standard deviations (SD) above the mean OD of the negative control; 2- and 4-folds values were used to discriminate between the other levels (low-to-medium = 0.115; medium-to-high = 0.23). For each isolate, the biofilm data are shown, and the corresponding level of biofilm formation is indicated.

Isolate	Biofilm	Level
**2-4**	0.061	Low
**7-2**	0.077	Low
**8-2**	0.239	High
**12-2**	0.771	High
**16-1**	0.07	Low
**17-1**	0.343	High
**20-1**	0.156	Medium
**23-1**	0.076	Low
